# Experimental and Numerical Impact Analysis of Automotive Bumper Brackets Made of 2D Triaxially Braided CFRP Composites

**DOI:** 10.3390/ma13163554

**Published:** 2020-08-12

**Authors:** Robert Böhm, Andreas Hornig, Tony Weber, Bernd Grüber, Maik Gude

**Affiliations:** 1 Faculty of Engineering, Leipzig University of Applied Sciences, Karl-Liebknecht-Straße 134, D-04277 Leipzig, Germany; robert.boehm.1@htwk-leipzig.de; 2 Institute of Lightweight Engineering and Polymer Technology (ILK), Technische Universität Dresden, 01307 Dresden, Germany; tony.weber@tu-dresden.de (T.W.); bernd.grueber@tu-dresden.de (B.G.); maik.gude@tu-dresden.de (M.G.)

**Keywords:** braided composites, impact, computational modelling, damage, drop tower testing

## Abstract

The impact behavior of carbon fiber epoxy bumper brackets reinforced with 2D biaxial and 2D triaxial braids was experimentally and numerically analyzed. For this purpose, a phenomenological damage model was modified and implemented as a user material in ABAQUS. It was hypothesized that all input parameters could be determined from a suitable high-speed test program. Therefore, novel impact test device was designed, developed and integrated into a drop tower. Drop tower tests with different impactor masses and impact velocities at different bumper bracket configurations were conducted to compare the numerically predicted deformation and damage behavior with experimental evidence. Good correlations between simulations and tests were found, both for the global structural deformation, including fracture, and local damage entities in the impact zone. It was proven that the developed phenomenological damage models can be fully applied for present-day industrial problems.

## 1. Introduction

Engineering with novel, light-weight, high-performance composite materials has been a focus of science and many different industrial sectors in recent years due to increasing requirements for the reduction of climate-damaging emissions and economizing the use of fossil energy sources. In this context, textile-reinforced composites offer enormous lightweight potential [1,2]. Beyond that, they feature highly specific stiffnesses and strengths [3,4], as well as a high energy absorption capabilities during crash and impact loading, and are therefore suitable for dynamically loaded, high-performance structures. Particular interest has been shown in braided composites because of their near net shape manufacturing flexibility and their relatively low manufacturing costs [5,6]. Today, braided composites are already being used in several structural components, especially in the automotive industry, e.g., as body structures [7], crash tubes [8] or bumper brackets [9].

To capitalize upon the advantageous properties of braided composites for impact and crash loaded applications, composite engineers still face some important challenges. Composite vehicle design is nowadays driven by advanced numerical simulation techniques. However, the commercial finite element codes which are mostly used in industry are not fully suitable to predict the complex deformation and fracture mechanisms of braided composites in particular since they are usually optimized to computing time, and not for correctness of damage prediction. For that reason, the development of suitable simulation techniques and material models for different types of textile reinforcement has been a major research field in recent years, see e.g., [10,11,12,13,14,15,16,17,18,19,20,21,22,23,24,25,26,27]. On the one hand, such methods have to take complex micro-meso-macro interactions within the material into account. On the other hand, they have to fulfill the requirements which arise from practically-oriented structural designs (numerical effort, short calculation time, practicability). A further problem appears because novel damage models are not implemented into commercial finite element codes in most cases.

The reliable determination of the input parameters at reasonable expense for such models is another key issue facing composite engineering [26]. Recent developments in the field of high-speed testing have shown that comprehensive characterization of the strain rate dependent material behavior of textile-reinforced composites is possible, although not all drawbacks are completely eliminated [27,28,29,30,31]. However, the strain rate dependent characterization of composites is still a subject of ongoing research, especially with regard to the out-of-plane behavior. This is mainly caused by the lack of availability of sufficiently thick and representative specimen materials [32]. For higher strain rates, the Split-Hopkinson-Bar test setup is commonly used to determine in-plane [33] and out-of-plane material characteristics [34]. Also, mixed experimental–numerical approaches based on multiscale modelling are now being employed to determine material characteristics [35].

To evaluate state-of-the-art numerical and experimental impact modelling, an automotive Carbon fiber reinforced plastic (CFRP) bumper bracket system was chosen. BMW decided to use such CFRP bumper brackets within the BMW M6 some years ago [9]. Nevertheless, the failure characteristics of the composite bumper brackets varied widely compared to those of their metallic predecessors, and are not yet completely understood, a fact which is responsible for a lot of economic costs [36]. In order to improve the lightweight potential of braided bumper brackets in the future, the scientific goals of this study are: (a) to describe the impact behavior (structural deformation, fracture, damage in the impact zone) using, for the first time, novel, specially adapted, phenomenological continuum damage mechanics (CDM) models that have been implemented into commercial finite element codes, (b) to characterize the impact performance of the bumper brackets experimentally by drop tower tests that were specially designed for this study, and (c) to evaluate the capabilities of the models, both with respect to mechanical correctness and practicability for industrial use. The study intends to prove whether or not a comprehensive crash and impact analysis of industrial CFRP components is possible under real-life conditions, considering not only subquestions of the design process, but also the overall “design picture”.

## 2. CFRP Bumper Brackets

**Figure 1:**
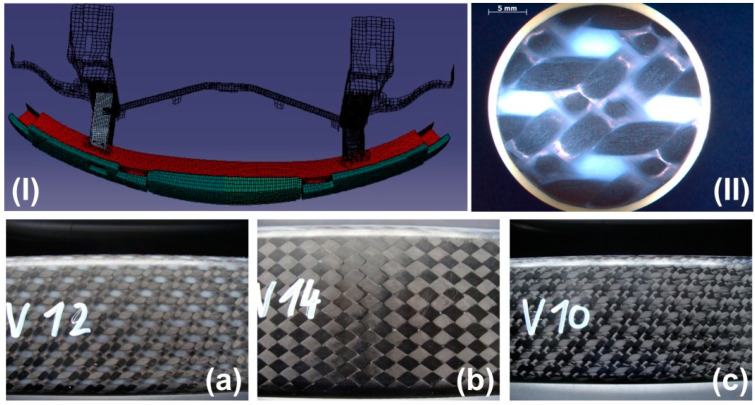
CFRP braided bumper bracket: (**I**) bumper bracket (red) with pedestrian collision protection (green) and crash tubes (behind the bumper bracket); (**II**) micrograph of the representative volume of the BMW serial configuration ±45° with 0° filler yarn; bumper bracket configurations: (**a**) 4 layers ±45° with 0° filler yarn; (**b**) 9 layers ±30°; (**c**) 4 layers ±45° with 0° filler yarn and unidirectional 0° layers between every braided layer.

In order to improve the driving dynamics of the BMW M6, BMW decided to replace the metallic front and rear bumper brackets with composite components [36]. The primary requirement of the braided bumper bracket is to withstand a frontal pedestrian impact at a velocity of approx. 20 km/h. The bumper bracket must not break and is designed to transfer the loading into the crash boxes where the impact energy is dissipated. The bumper brackets are manufactured with braiding technology at Kümpers Composites GmbH & Co. KG (Rheine, Germany) and delivered to BMW on sand cores. Consolidation is performed using RTM technology with EPR4695 epoxy resin (Bakelite) and EPH5357 hardener. Figure 1 shows a finite element model of the bumper bracket. The bumper bracket (red) is mounted to the crash boxes (behind the bumper bracket). In front of the bumper bracket, the pedestrian collision protection (green) is attached.

Three different material configurations were analyzed in this study: the BMW serial configuration with four 2D triaxially braided ±45° layers with 0° filler yarn (configuration (a)), a bumper bracket with lower axial stiffness (configuration (b)) and a bumper bracket with higher axial stiffness (configuration (c)); see Figure 1. Configuration (b) consists of nine 2D biaxially braided ±30° layers. In configuration (c), unidirectional 0° layers are added into the standard configuration (a) between every braided layer. Tenax STS 5631 fibers are used as braiding yarns and Zoltek Panex35 continuous tows for the filler yarns. The 2D biaxial braids are designed in a way that full coverage is achieved (see Figure 1b). Full coverage does not occur with the 2D triaxial braids, which causes relatively large resin-rich areas of approx. the width of a filler yarn (see Figure 1a). The thickness of each layer results from the homogeneous distribution of the braided preforms over the constant bumper bracket thickness of 10 mm. Fiber volume contents of 52% for configuration (a), 53% for configuration (b) and 61% for configuration (c) were measured.

Within this study, the crash boxes and pedestrian collision protection were not analyzed because the main objective of the study was to evaluate the ability of damage models to describe the mechanical response of braided composite structures, and not to study the complex interactions between different automotive components.

## 3. Drop Tower Experiments

**Figure 2:**
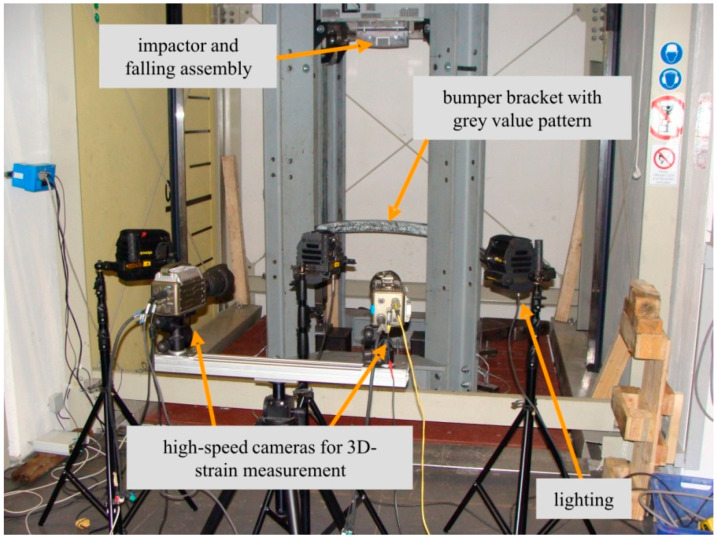
Drop tower test setup.

Frontal pedestrian impact was experimentally reproduced by impact tests using a 27-m high drop tower. Different impactor masses and impact velocities were used for all three bumper bracket configurations. All drop tower tests made use of a high-speed camera measurement system. Figure 2 shows the newly developed drop tower setup. 

**Figure 3:**
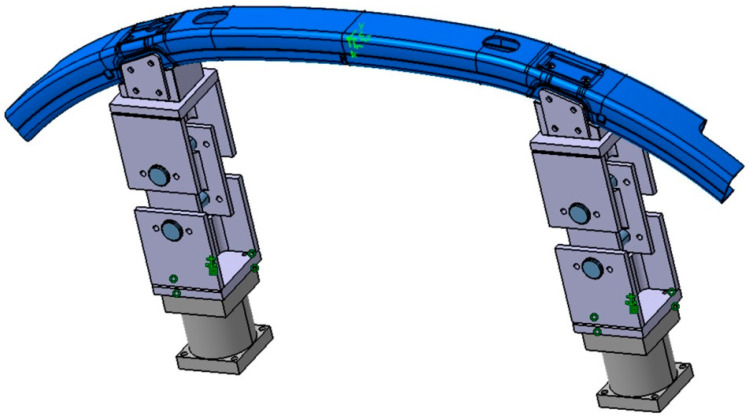
Clamping of the bumper bracket for the drop tower tests: solid bearing (**left**) and articulated bearing (**right**) with load cells.

The quality of the test results under highly dynamic loading conditions strongly relies on the synchronous triggering of all relevant measurement equipment. The synchronicity is thereby related both to the internal clients of a single measurement system and to different measurement systems to each other. Within the drop tower tests, two high-speed cameras and two individual load cells were used: one at the solid bearing and the other at the articulated bearing of the bumper bracket (Figure 3). The setup with one solid bearing and one articulated bearing was chosen in order to guarantee a test situation with the greatest possible comparability to the standard BMW crash test. Preliminary tests at different test facilities have shown that a test situation with two solid bearings creates too much damage in the clamping area of the bumper brackets. The temporal synchronism of the force components was ensured by a hardware system which was specially matched to impact and crash tests. A central controller within the hardware system regulated the synchronicity of the connected load cells and delivered the force data with a time stamp to the evaluation unit. To ensure the synchronicity of the two cameras, an internal trigger mode of the cameras was used. Upon initiation of an external trigger, the camera in master-mode transmitted a cue to the slave-mode camera. The external triggering was realized by a height-adjustable light barrier. Therewith, the required trigger signals for the instrumentation were generated in combination with a trigger controller.

**Figure 4:**
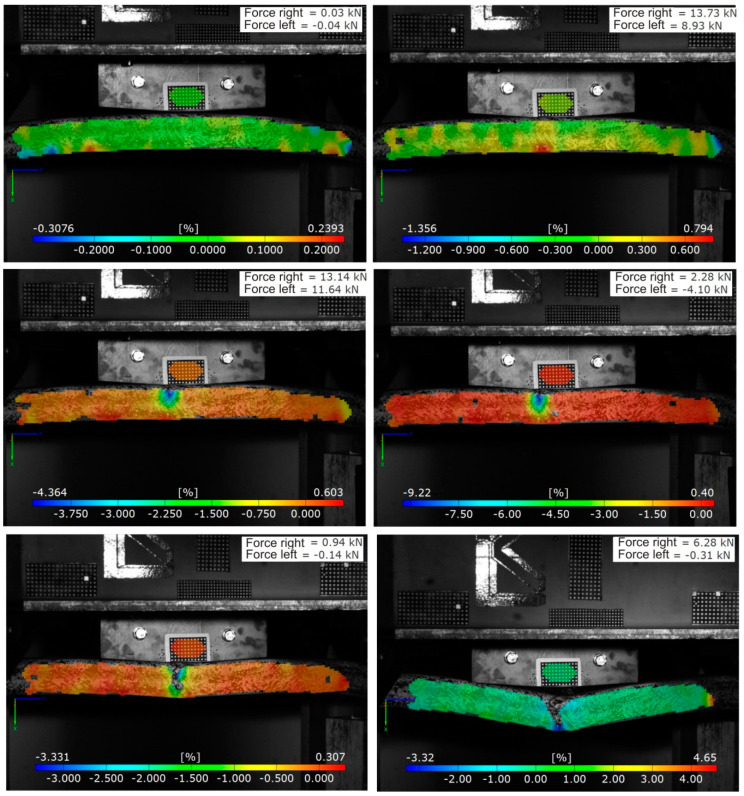
Deformation characteristics of bumper bracket configuration (a), layup: 4 layers ±45° with 0° filler yarn (2D triaxial braid), during a drop tower test with m_imp_ = 435 kg and v_imp_ = 8 km/h.

The temporal resolution of the force measurement was chosen to be 20 kHz in a measuring range from 20 kN to 500 kN. The minimal time step of the cameras depended on the desired resolution. In this study, a resolution of 800 × 600 pixels was used, yielding a recording rate of up to 6600 fps. The used lighting allowed a shutter speed of approx. 100 s, which is acceptable for velocities up to 10 m/s. Both cameras had to be calibrated relative to the measurement volume in order to continue to use the recorded video material as a foundation for digital image correlation. Within the calibration, the distance and the angle of both Charge-coupled Device (CCD) sensors to each other could be additionally determined. Based on a triangulation, the used software was able to calculate the deformation field of the test object. In combination with the associated time stamp, the velocities and accelerations of the objects could additionally be determined. The degree of detail of the optical evaluation was defined by the resolution of the camera relative to the considered measurement volume. A phenomenological damage analysis of the bumper brackets subjected to impact required a coupled evaluation of video data and measured mechanical values. Figure 4 shows an example of such a phenomenological impact analysis using the axial strain and the recorded forces at the bearing as characteristic mechanical values. Such an evaluation was possible for every externally recorded measurement value of the drop tower test which exhibited a time stamp that coincided with the time stamp of the video data. When the pairs of values did not coincide relative to the time stamp, interpolation was also possible with the used software. Beyond the camera-based optical-mechanical data evaluation, all tested bumper brackets were analyzed with respect to the damage which occurred in the impact zone by different nondestructive testing methods (ultrasonics, computer tomography). Two impactor masses in combination with three impact velocities were experimentally investigated. Slightly bent steel impactors with m_imp_ = 435 kg and m_imp_ = 154 kg were used to prevent strongly localized damage phenomena; see Figure 4. The length of the impactor was set to approx. 40% of the bumper bracket length. The impact velocity was adjusted by choosing three different drop heights: 1.00 m, 0.25 m and 0.10 m. In so doing, impact velocities of 16 km/h, 8 km/h and 5 km/h were analyzed. A complete video of a drop tower test (m_imp_ = 435 kg, v_imp_ = 16 km/h, configuration (a)) is given in the Supplementary Materials.

All of the performed impact experiments followed the same general phenomenology: after first contact of the impactor with the bumper bracket (Figure 4, top left), a strain increase on the bottom of the bumper bracket (Figure 4, top right), and thus also a stress concentration in the tensile domain, was observed. However, local damage initiation was always observed on the top of the bumper bracket in regions of compression (Figure 4, middle left). Cracks then started to propagate through the lateral parts of the bumper bracket (Figure 4, middle right) until reaching the bottom, at which time the bumper bracket was fully cracked (Figure 4, bottom left). Afterwards, a strong deformation increase was observed without any major damage increase in the structure (Figure 4, bottom right). For parameter setups with lower impactor mass (m_imp_ = 154 kg) and/or lower impact velocities (v_imp_ = 8 km/h and v_imp_ = 5 km/h), the behavior was observed to be similar but less pronounced. A full crack through the structure was then not observed in any case.

## 4. Numerical Impact Modelling

### 4.1. Material Model and Input Data

The continuum damage mechanics (CDM) model published in [12] was used in this study. The constitutive equation for the damaged biaxial or 2D triaxial braided layer was defined via the compliance matrix $\tilde{S}$, considering orthotropic damage according to the classical principles of CDM:

(1) $$\tilde{S}=\left[\begin{array}{ccc} \frac{1}{E_{1}\left(1-D_{1}\right)} & -\frac{\nu_{12}}{E_{2}\left(1-D_{2}\right)} & 0\\ -\frac{\nu_{21}}{E_{1}\left(1-D_{1}\right)} & \frac{1}{E_{2}\left(1-D_{2}\right)} & 0\\ 0 & 0 & \frac{1}{2G_{12}\left(1-D_{6}\right)} \end{array}\right].$$

Damage parameters D_1_ and D_2_ define damage in the axes of orthotropy, while damage parameter D_6_ defines damage due to shear. Both 2D biaxially and 2D triaxially braided composites fulfil the definition of orthotropy as long as no unsymmetric fiber rearrangement occurs during loading. The experimental results reported in [37] reveal only slight nonlinearities when the braided layer was loaded in the fiber direction. However, pronounced nonlinear behavior was observed for off-axis loading and shear. Another characteristic observation for the latter load cases was the onset of damage already at low stresses. The stress-strain formulation for the damaged layer eventually became:

(2) $$\sigma_{i}={\tilde{Q}}_{ij}\varepsilon_{j}$$

with

(3) $${\tilde{Q}}_{ij}=\left[\begin{array}{ccc} {\tilde{Q}}_{11} & {\tilde{Q}}_{12} & 0\\ {\tilde{Q}}_{21} & {\tilde{Q}}_{22} & 0\\ 0 & 0 & {\tilde{Q}}_{66} \end{array}\right]$$

and

(4) $${\tilde{Q}}_{11}=\left(1-D_{1}\right)\frac{E_{1}}{1-\nu_{12}\nu_{21}},\;{\tilde{Q}}_{22}=\left(1-D_{2}\right)\frac{E_{2}}{1-\nu_{12}\nu_{21}},\;{\tilde{Q}}_{66}=\left(1-D_{6}\right)\;\;G_{12},\;{\tilde{Q}}_{12}={\tilde{Q}}_{21}=\left(1-D_{6}\right)\frac{\nu_{21}E_{2}}{1-\nu_{12}\nu_{21}}-D_{6}\sqrt{\left(1-D_{1}\right)\left(1-D_{2}\right)\frac{E_{1}E_{2}}{{\left(1-\nu_{12}\nu_{21}\right)}^{2}}}.$$

The onset of damage and total failure were modelled using the stress-based failure criteria of Cuntze; see [12]. Damage evolution laws of type

(5) $$\varphi^{j}=\tanh\left[\;\beta_{j}\left(s_{j}-s_{j0}\right){\;}^{\kappa_{j}}\right]$$

were used to describe the evolution of the damage parameters $D_{i}=\varphi^{j}q_{i}$. For each of the Cuntze fracture modes *j*, a damage growth parameter *φ_j_* was calculated from (5) using two free model parameters, i.e., *β_j_* and *κ_j_*. The parameter *s_j_* is the damage threshold function according to the Cuntze-type formulation in [12]. The parameter *s_j_*_0_ was used to model multiaxial stress states, and is not a free material parameter. As discussed in [29], strain rate dependent stiffness and strength values were modelled using the Johnson-Cook [38] approach:

(6) $$E_{i}\left({\dot{\varepsilon }}_{i}\right)=E_{i}^{\left(ref\right)}\left[1+A_{i}^{E}\ln\left(\frac{{\dot{\varepsilon }}_{i}}{{\dot{\varepsilon }}_{i}^{\left(ref\right)}}\right)\right],\;R_{i}^{0}\left({\dot{\varepsilon }}_{i}\right)=R_{i}^{\left(0,ref\right)}\left[1+A_{i}^{0}\ln\left(\frac{{\dot{\varepsilon }}_{i}}{{\dot{\varepsilon }}_{i}^{\left(ref\right)}}\right)\right],\;R_{i}^{}\left({\dot{\varepsilon }}_{i}\right)=R_{i}^{\left(ref\right)}\left[1+A_{i}^{R}\ln\left(\frac{{\dot{\varepsilon }}_{i}}{{\dot{\varepsilon }}_{i}^{\left(ref\right)}}\right)\right],$$

where $E_{i}\left({\dot{\varepsilon }}_{i}\right)$, $R_{i}^{0}\left({\dot{\varepsilon }}_{i}\right)$ and $R_{i}^{}\left({\dot{\varepsilon }}_{i}\right)$ are the strain rate dependent basic engineering constants, onset of damage (in terms of stress) and strengths, respectively. Only one additional material parameter per equation (A) was used to determine the strain rate dependency. Experimental parameter identification using high-speed tensile tests was performed on flat specimens of all three investigated configurations. Table 1 and Table 2 summarize the material and model parameters necessary to capture the nonlinear failure behavior of the two different types of braids used in the bumper bracket configurations: 2D triaxially braided ±45° layers with 0° filler yarn (used in configuration (a) and (c)) and 2D biaxially braided ±30° layers (used in configuration (b)). 

**Figure 5:**
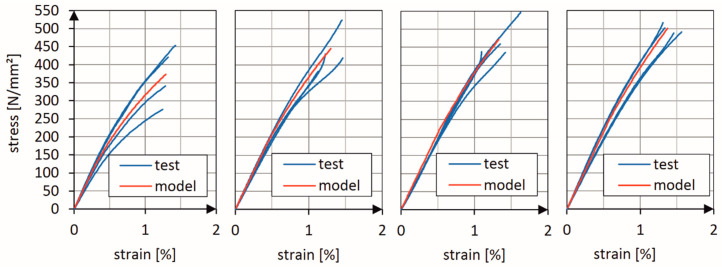
Comparison of measured and predicted stress-strain curves for material configuration (a), layup: 4 layers ±45° with 0° filler yarn (2D triaxial braid), for four different strain rates: 2 mm/min, 10 mm/s, 100 mm/s and 1000 mm/s (from left to right).

**Figure 6:**
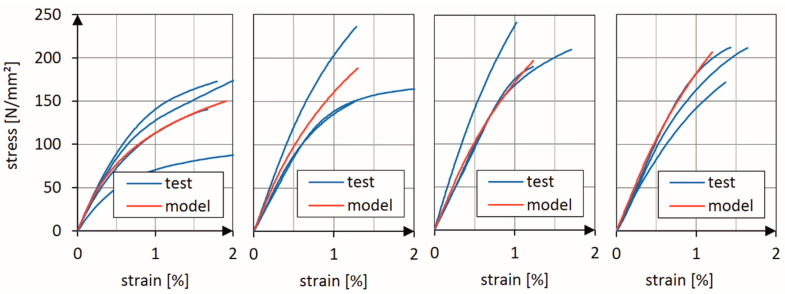
Comparison of measured and predicted stress-strain curves for material configuration (b), layup: 9 layers ±30° (2D biaxial braid), for four different strain rates: 2 mm/min, 10 mm/s, 100 mm/s and 1000 mm/s (from left to right).

Figure 5 (2D triaxially braided ±45° layers with 0° filler yarn) and Figure 6 (2D biaxially braided ±30° layers) show a comparison between the stress-strain curves which were experimentally determined in the basic tests and the stress-strain curves predicted by the calibrated model. Four different strain rates (2 mm/min, 10 mm/s, 100 mm/s and 1,000 mm/s) were used for model calibration. The material data for simple unidirectional layers were taken from BMW’s material database. It is obvious that the damage model was able to correctly predict the nonlinear behavior of both the 2D biaxial and 2D triaxial braids.

### 4.2. Implementation

**Figure 7:**
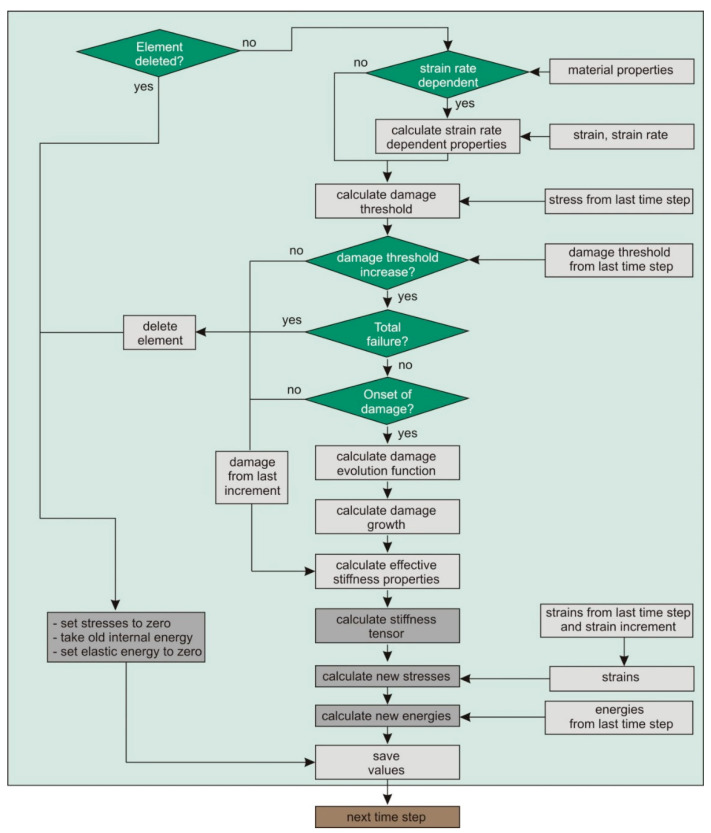
Flowchart of the VUMAT damage calculation within one time step.

The used CDM model [12] was implemented as a user defined consitutive model (VUMAT) into Abaqus/Explicit, and could be used both for shell (2D implementation) and volume elements (3D implementation). The subroutine was called at the beginning of each time step and used solution-dependent state variables (SDV), whereby one SDV regulated element deletion (when total failure was calculated by the damage model). Figure 7 schematically shows the calculation flow of the VUMAT within one time step.

### 4.3. Simulation of the Drop Tower Tests

**Figure 8:**
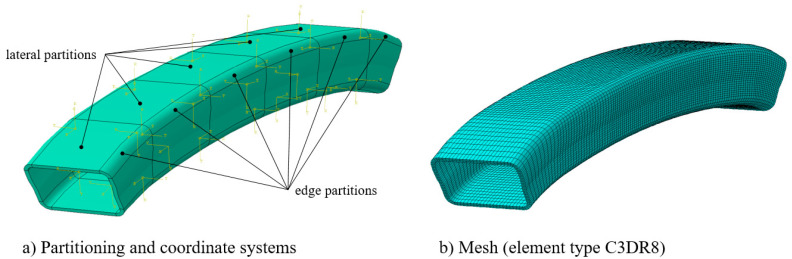
Finite Element (FE) model: (**a**) partitioning and (**b**) meshing.

All configurations (six load cases (i.e., two impactor masses, three impact velocities) and three different bumper bracket configurations) were numerically investigated using the implemented VUMAT and volume elements (element type C3DR8). The bumper bracket model was subdivided into six parts in terms of length and eight parts in terms of circumference. In total, 48 partitions arose: 24 for the lateral surfaces (Cartesian coordinate system) and 24 for the edge regions with their own cylindrical coordinate systems (Figure 8). Four elements in the thickness direction were used to accurately predict the stress distribution and to capture successive failure mechanisms in all three directions. Element type C3DR8 was used. In total, this resulted in 45,600 elements (57,500 nodes) with an average element length (axial) of 7.4 mm, width (circumferential) of 2.7 mm and thickness of 1.1 mm.

**Figure 9:**
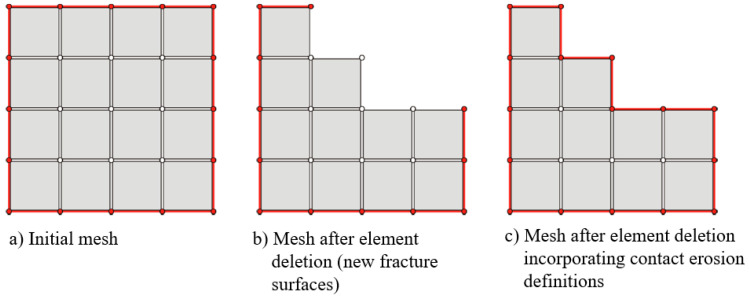
Effects of element deletion on the contact area (red line) of a mesh.

The contact between impactor and bumper bracket was realized by the general contact option (all with self). Because elements were deleted during the impact process when total failure was indicated (see Figure 9), the contact area had to be regenerated by taking the newly established fracture surfaces into account and avoiding mesh penetrations. (contact erosion issue). “Surferode” surfaces were generated, containing all initial external surfaces and all internal element surfaces (Figure 9). The constraint of the bumper bracket was realised at the element nodes which were connected to the structure via multipoint constraints. The impactor was modelled as a rigid body.

## 5. Results and Discussion

**Figure 10:**
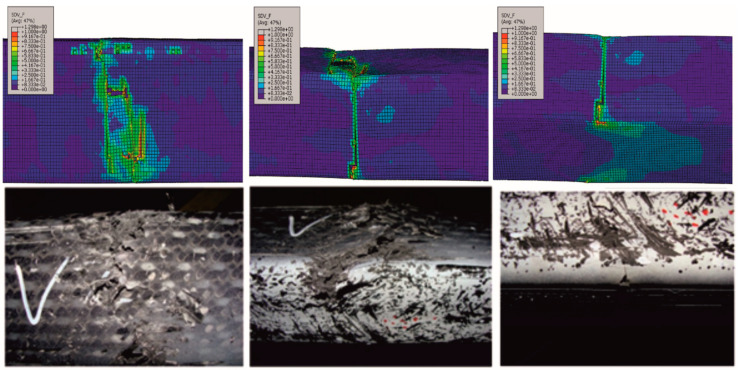
Comparison of the fracture phenomena between simulation and drop tower test (bumper bracket configuration (a), layup: 4 layers ±45° with 0° filler yarn (2D triaxial braid), m_imp_ = 435 kg, v_imp_ = 1 6 km/h): front-side (**left**), lateral surface (**middle**) and backside (**right**).

Figure 10 shows a comparison of the simulated fracture phenomena (shown in the FE pictures: damage threshold/stress effort) and pictures of the broken bumper bracket after the experiment. The separately shown pictures of the front, lateral and back side of the bumper bracket illustrate that the damage phenomenology of the bumper bracket reinforced with both 2D biaxial braids and 2D triaxial braids can be reproduced significantly well. The crack patterns in the backside were almost identical. The characteristic break-out on the edge of lateral side and front-side (shown in Figure 10 left) occurred in the test as well as in the simulation.

Beyond that, the crack propagation can be better analyzed in the simulation because of the higher temporal resolution. By this means, it was numerically shown that first damage was initiated at the front edges; see also Figure 4. Starting from those edges, the cracks propagated into the front and the lateral sides. This damage progress was very plausible due to the high stresses which occurred at the edges because of the high level of structural stiffnesses.

**Figure 11:**
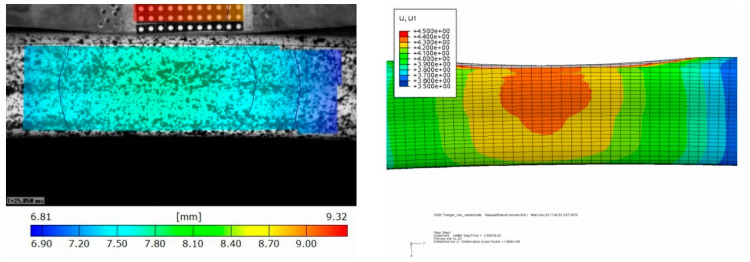

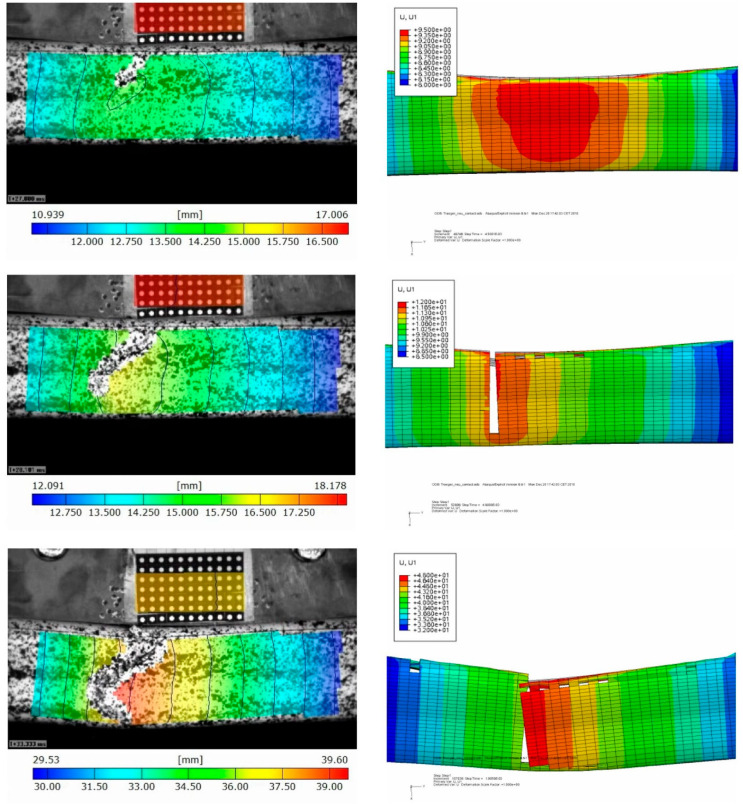
Comparison of experimentally observed (**left**) and numerically predicted (**right**) deformations of bumper bracket configuration (b), layup: 9 layers ±30° (2D biaxial braid), with m_imp_ = 154 kg and v_imp_ = 16 km/h: 2.5 μs (edge failure), 4.5 μs (crack initiation in lateral surfaces), 4.8 μs (crack propagation) and 10 μs (total failure).

**Figure 12:**
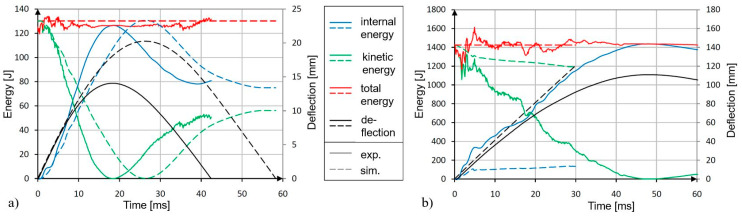
Comparison of experimental and numerically determined deformations and energies of bumper bracket configuration (b), layup: 9 layers ±30° (2D biaxial braid): (**a**) 5 km/h (drop height 0.1 m), (**b**) 16 km/h (drop height 1 m).

Beside the correct representation of the damage phenomenology, the structural deformation of the bumper brackets was analyzed, as was the velocity profile of the impactor. A comparison of the simulated bumper bracket deformation with the video data that was generated by the evaluation of the greyscale correlation method data is shown in Figure 11. It becomes obvious that the simulation predicted slightly higher deformations for the drop tower test (except after the bumper bracket was fully cracked). The predicted damaged zones were slightly larger. Therefore, elements were gradually deleted which decreased the structural stiffness of the bumper bracket. Thus, higher deformations were predicted. These findings were confirmed by comparing the experimentally and numerically determined deformation and energy plots over time (Figure 12). To reduce this effect, the underlying damage model [12] will have to be modified in the future in order to describe the softening behaviour of the material after exceeding the strength in a better way. Existing advanced viscoelastic-plastic damage models like [25] may be candidate models as well. In general, quantitative agreement between simulation and test was better at lower the impact energies; nonetheless, sufficient qualitative agreement was achieved for all cases.

## 6. Conclusions

In this experimental–numerical study, it was shown that novel phenomenological damage models for textile-reinforced composites are well suited to industrial applications of high complexity. In this case, bumper brackets made of 2D biaxially and 2D triaxially braided composites loaded by impact were analyzed. With the provided results, engineers in the lightweight industry are in a position to use advanced models to design textile composites, instead of standard models that are mostly based on simulating unidirectional plies. Thus, considerably more reliable impact designs and damage evaluations are possible for textile-reinforced components. However, some limitations need to be considered: notably, strain rate dependent failure in the thickness direction of the textile composite was not fully experimentally verified due to the lack of testing methods. Therefore, accurate evaluation of complex 3D stress states is still difficult. The applied material model was designed to be used for one-time impact loads. Viscoplastic effects were, therefore, not considered yet, but could be easily incorporated, e.g., by using advanced viscoelastic-plastic damage models, as in [25].

## Figures and Tables

**Table 1:** Engineering constants, onset of damage and strengths.

Material Parameter	Unit	Configuration: (a), (c) Layup: ±45°/0°	Configuration: (b) Layup: ±30°
$E_{1}$	[GPa]	40.9	21.2
$E_{2}$	[GPa]	8.0	7.9
$G_{12}$	[GPa]	6.7	16.0
$\nu_{12}$	[–]	0.83	1.30
$R_{1t}^{0}$	[MPa]	118	24
$R_{1c}^{0}$	[MPa]	100	24
$R_{1t}^{}$	[MPa]	373	165
$R_{1c}^{}$	[MPa]	305	165
$R_{2t}^{0}$	[MPa]	50	24
$R_{2c}^{0}$	[MPa]	50	49
$R_{2t}^{}$	[MPa]	150	51
$R_{2c}^{}$	[MPa]	150	97
$R_{12}^{0}$	[MPa]	10	10
$R_{12}^{}$	[MPa]	130	80

**Table 2:** Strain rate parameters and model parameters.

Model Parameter	Unit	Configuration: (a), (c) Layup: ±45°/0°	Configuration: (b) Layup: ±30°
${\dot{\varepsilon }}_{1}^{\left(ref\right)}$	[1/s]	0.00015	0.00033
${\dot{\varepsilon }}_{2}^{\left(ref\right)}$	[1/s]	0.00037	0.00030
${\dot{\gamma }}_{12}^{\left(ref\right)}$	[1/s]	0.00030	0.00030
$\beta_{1}^{t}$	[–]	0.14	0.12
$\kappa_{1}^{t}$	[–]	1.00	1.10
$\beta_{1}^{c}$	[–]	0.14	0.12
$\kappa_{1}^{c}$	[–]	1.00	1.10
$\beta_{2}^{t}$	[–]	0.09	0.43
$\kappa_{2}^{t}$	[–]	1.50	1.50
$\beta_{2}^{c}$	[–]	0.09	0.43
$\kappa_{2}^{c}$	[–]	1.50	1.50
$\beta_{12}^{}$	[–]	1.00	1.00
$\kappa_{12}^{}$	[–]	1.00	1.00
$A_{1}^{E}$	[–]	0.00427	0.00450
$A_{1}^{0t}$	[–]	0.10077	0.19410
$A_{1}^{0c}$	[–]	0.10077	0.19410
$A_{1}^{t}$	[–]	0.03170	0.02290
$A_{1}^{c}$	[–]	0.03170	0.02290
$A_{2}^{E}$	[–]	0.02577	0.02740
$A_{2}^{0t}$	[–]	0.09978	0.05630
$A_{2}^{0c}$	[–]	0.09978	0.05630
$A_{2}^{t}$	[–]	0.03574	0.01900
$A_{2}^{c}$	[–]	0.03574	0.01900
$A_{12}^{E}=A_{12}^{0}=A_{12}^{}$	[–]	0	0

## Supplementary Materials

The following are available online at https://www.mdpi.com/1996-1944/13/16/3554/s1, Video S1: Deformation characteristics of bumper bracket configuration.
